# Perceptions of Faculty and Students About Use of Artificial Intelligence in Medical Education: A Qualitative Study

**DOI:** 10.7759/cureus.57605

**Published:** 2024-04-04

**Authors:** Sarah M Salih

**Affiliations:** 1 Department of Community and Family Medicine, Faculty of Medicine, Jazan University, Jazan, SAU

**Keywords:** chat gpt, ethical concerns, learning, knowledge, research, assessment, academic activities, medical education, artificial intelligence

## Abstract

Background: Artificial intelligence (AI) implies using a computer to model intelligent behavior with minimal human intervention. With the advances of AI use in healthcare comes the need to reform medical education to produce doctors competent in AI use. Therefore, this qualitative study was conducted to explore faculty and students' perspectives on AI, their use of AI applications, and their perspective on its value and impact on medical education at a Saudi faculty of medicine.

Methods: This qualitative study was conducted at the Faculty of Medicine, Jazan University in Saudi Arabia. A direct interview was held with 11 faculty members, and six focus group discussions were conducted with students from the second to sixth year (34 students). Data were collected using semi-structured open-ended interview questions based on relevant literature.

Findings: Most respondents (91.11%) believed AI systems would positively impact medical education, especially in research, knowledge gain, assessment, and simulation. However, ethical concerns were raised about threats to academic integrity, plagiarism, privacy/confidentiality issues, and AI's lacking cultural sensitivity. Faculty and students felt a need for training on AI use (80%) and that the curriculum could adapt to integrate AI (64.44%), though resources were seen as currently needing to be improved.

Conclusion: AI's potential to enhance medical education is generally viewed positively in the study, but ethical concerns must be addressed. Integrating AI into medical education programs requires adequate resources, training, and curriculum adaptation. There is still a need for further research in this area to develop comprehensive strategies.

## Introduction

Artificial intelligence (AI) can be defined generally as a broad concept, which is challenging. However, it conveys the idea of using a computer to model intelligent behavior with minimal human intervention [1]. AI refers to the capacity of digital computers or robots that can be controlled using a computer to perform functions that intelligent beings usually do. The term AI is often used to denote the effort of constructing devices and objects capable of thinking within the same cognitive framework in which humans perform their mental processes, such as the ability to reason, discover meaning, generalize, or learn from experience [2].

AI has evolved since it was first described around 1956 [3] and continues to develop rapidly, especially in the post-coronavirus disease 2019 (COVID-19) era. It has touched many aspects of our lives, and healthcare is no exception. With the advances of AI use in healthcare comes the need to reform medical education to produce doctors competent in AI use [4].

In 2019, the Standing Committee of European Doctors (CPME), on its perspective on the use of AI in healthcare, stressed that "it is important to base AI development in healthcare on robust evidence; its use must be accountable, non-discriminatory and respect patients' privacy" [5].

AI can significantly add value to medical education, especially in learning support and assessment [6]. For example, it can help guide student training and problem-solving training and provide standardized evaluation and simulation [6]. However, challenges and concerns exist, especially regarding ethics and reliability [7]. 

Literature has shown that medical students generally have a positive attitude towards AI and about being trained on its use in different aspects of medical education and healthcare [8,9]. In a survey of Canadian healthcare students, most students revealed AI would affect their careers within the next decade [9]. Almost all students of the Faculty of Medicine at Kuwait University, Kuwait, see AI in their profession as a beneficial thing and think students must receive formal AI training [10]. Another study carried out with the students of Riyadh, Saudi Arabia, observed a positive attitude of pharmacy aspirants towards AI and the need for education and training in AI technology [11]. Saudi Arabian dentists and professionals expressed their strong belief in AI use as well as a positive attitude. In addition, they showed a desire to take professional courses in AI for better use of its technologies [12].

Medical students must be well-trained and technically ready to practice healthcare in technologically advanced AI-enabled healthcare settings [4]. However, AI integration within medical curricula is still shy, fragmented, and inconsistent [7]. Therefore, this qualitative study was conducted to explore faculty and students' perspectives on AI, their use of AI applications, and their perspective on its value and impact on medical education at a Saudi faculty of medicine.

## Materials and methods

This was a qualitative case study conducted at the Faculty of Medicine, Jazan University, Jazan, Saudi Arabia, from November 2023 to February 2024. The total number of faculty members in the university is 117 (89 male and 28 female); 76 are Saudi nationals. The total number of registered medical students for 2023-2024 is 901 (442 females and 459 males).

A direct interview was conducted with 11 faculty members who were selected by convenience sampling. They represent different academic ranks and different departments. All interviews took place at the office of the faculty member. Focus group discussions were conducted with students from the second to sixth year (34 students). Student selection was achieved by sending an invitation to the focus group discussions through the class leaders. Students who showed up at the meeting were included in the study. First-year medical students study at the preparatory college and are not within the Faculty of Medicine. Six focus group discussions were conducted with students by two trained faculty members. The two researchers limited the number to six meetings after saturation was reached by the fifth focus group discussion. The meetings took place at different teaching halls at the faculty and were audio recorded. 

Data were collected using semi-structured open-ended interview questions. Questions were based on relevant literature on the topic [10-13] to achieve the study objectives. Interviews and focus group discussions were documented, and transcripts were aggregated into a single Excel extraction sheet (Microsoft Corporation, Redmond, Washington, United States). 

Data analysis included frequencies and percentages for quantitative data, such as demographic information, and thematic analysis for qualitative data. A reflexive diary was used which helped in the stages of coding and finalizing the thematic analysis that was performed by two researchers. Some of the qualitative data was aggregated to find consensus on specific issues; therefore, using the transcripts some percentages were calculated. For example, when candidates were asked if they think resources are available to use AI, any participant who indicated a positive response like “yes” or “absolutely” was counted as yes, while negative responses like “no” or “disagree” were counted as no. No response or “I don’t know” or “not sure” were not counted.

Ethical clearance was obtained from the Standing Committee for Scientific Research at Jazan University (approval number REC-45/05/876).

## Results

A total of 45 subjects participated in the study; 11 were faculty members (24.44%) and 34 (75.56%) were medical students from the second to the sixth year. Twenty-three (51.11%) participants were males and 22 (48.89%) were females. The mean age for faculty members was 48.5 years, and for students, it was 22.6 years (range 21-55 years). Table 1 provides details of the demographic data of participants, their use of AI, and the types of AI most frequently used.

**Table 1:** Demographic data of participants, their use of AI, and types of AI most frequently used AI: artificial intelligence

Character	Faculty members	Students	Both groups
Male	7 (15.56%)	16 (35.55%)	23 (51.11%)
Females	4 (8.88%)	18 (40%)	22 (48.89%)
Total number	11 (24.44%)	34 (75.56%)	45 (100%)
Mean age in years	48.5 (range 35 to 55)	22.6 (Range 21 to 24)	35.55 (range 21 to 55)
Rank/Academic year	Lecturer: 1 (9%)	2nd year: 3 (8.82%)	Not Applicable
	Assistant professor: 7 (64%)	3rd year: 9 (26.47%)	
	Associate Professor: 2 (18%)	4th year: 9 (26.47%)	
	Professor: 1 (9%)	5th year: 4 (11.76%)	
		6th year: 9 (26.47%)	
Do you have an AI application on your phone/ tablet			
Yes	9 (81.8%)	29 (85.3%)	38 (84.4%)
No	2 (18.2%)	5 (14.7%)	7 (15.6 %)
Name of AI application most frequently used			
ChatGPT (Chat Generative Pre-trained Transformer)	7 (28%)	18 (72 %)	25 (65.8%)
POE (Platform for Open Exploration)	2 (22.2%)	7 (77.8%)	9 (23.7%)
Others	0 (0%)	4 (22.2%)	4 (10.5%)
Total	9 (23.7%)	29 (76.3%)	38 (100%)

For the sake of convenience of the study participants, direct interview was conducted with faculty members and focus group discussions for the students. The academic ranks of faculty members included one Lecturer (9%), seven (64%) Assistant Professors, two (18%) Associate Professors, and one (9%) Professor. They represented the following departments: Anatomy, Family and Community Medicine, Medicine, Obstetrics & Gynecology, Orthopedics, Pediatrics, and Surgery. Among the faculty members interviewed was a former dean of the faculty and the current head of the medical education unit. Six focus group discussions were conducted; the two researchers who conducted them limited the number to six meetings after saturation was reached by the fifh focus group discussion. The number of medical students who participated in these focus group discussions was three (8.82%) from the second year, nine (26.47%) from the third year, nine (26.47%) from the fourth year, four (11.76%) from the fifth year, and nine (26.47%) from the sixth year. First-year medical students were not interviewed because they are part of the preparatory college and outside the Faculty of Medicine. 

Regarding AI applications, 38 (84.4%) used AI applications, and seven (15.6 %) did not. The most commonly used AI application is ChatGPT (Chat Generative Pre-trained Transformer) by 25 (65.8%), followed by POE (Platform for Open Exploration), an online platform that allows accessing multiple chatbots, used by nine (23.7%) and other applications used by four (10.5%). However, about 10 respondents (26.3%) used more than one AI application. 

Regarding the use of AI in daily academic activities, faculty members used it mainly to construct exam questions, research ideas, brainstorm in general, and correct language and grammar. On the other hand, students primarily used them to find answers and information, as one student mentioned “You can ask any question instead of previously using Google”, and also to summarize, look for resources for research, and do assignments.

The reasons for using AI applications were the same for faculty members and students: to save time, practicality, easy-to-use structure, and extensive information pool. One respondent quoted: "Can do more than one request at a time". A unique use mentioned by a student was helping to memorize medical nomenclature and terminologies. 

Thematic analysis

Thematic analysis of qualitative data that emerged from the discussions was done by two researchers using a reflexive diary and coding. Initially, five themes were recognized and then these were aggregated into three major themes: (i) Faculty and students mostly hold a positive perspective on the value of AI and its impact on medical education; (ii) Faculty and students raised ethical concerns about the use of AI in medical education; (iii) Faculty and students have different perspectives on resource availability, curriculum adaptability, and the need for training to incorporate AI in medical education at Jazan University.

Table 2 summarizes themes that emerged from qualitative data and common responses related to them.

**Table 2:** Themes that emerged from qualitative data and common responses related to them

Theme	Common responses related to theme	
	Faculty members	Students
Faculty and students mostly hold a positive perspective on the value of AI and its impact on medical education	It will have a positive impact on medical education; A source of literature and an extensive information pool for research; Standardization of evaluation; Helpful in active learning, MCQs construction, scenario generation, training organization, and case simulation; Brainstorming in general; Saves time, quick, and easy-to-use AI would aid specialties like doing patient triage, radiology, and pathology. It cannot replace a specialty, needs human supervision & clinical sense is important	It will have a positive impact on medical education; Find information: answering questions, elaboration, explaining complex information like pathophysiology of disease and clinical terms; Used in summarization; Students can perform clinical procedures in a safe environment; Prepare assignments, tasks, and presentations; Save time, efficient & make things easier; A large information pool; Maybe aid pathologists, radiologists in patient care. It will help but not replace physicians, it’s a machine
Faculty and students raised ethical concerns about the use of AI in medical education	Not a trusted source, you need to review its work; Gives vague references; A threat to academic integrity, privacy, copyright issues; No permissions, no citation of sources, intellectual problems, and authorship issues; Depends on western values and cultures.	Plagiarism, not using own thoughts; Information inaccuracy; Ownership issues, no respect for intellectual property, and conflict of interests; Questionable interpretation of information; Privacy and confidentiality issues; Needs human supervision.
Faculty and students have different perspectives on resource availability, curriculum adaptability, and the need for training to incorporate AI in medical education at Jazan University	Resources are available; The faculty need to invest in this technology; There is a need for human resource experts in the field; There is a need for software & updates; Curriculum can adapt and no reform needed; Might need some modification in course description, some regulation & adaptation.	Resources are fine; AI is inexpensive and widely available but how to invest in this technology; Faculty members need training on AI use; Curriculum can probably adapt.

Faculty and Students Mostly Hold a Positive Perspective on the Value of AI and its Impact on Medical Education

As a previous dean mentioned, “AI is an asset in every aspect of education”. Most respondents (n=41, 91.11%) had the view that AI systems would have a positive impact on medical education. They highlighted the advantages of using AI in research, knowledge gain, assessment, and simulation. As one faculty member notes: “It can be useful to switch to computerized exams,” referring to the current paper-based examination procedures at the faculty. Another faculty member specified standardized patient simulation as an asset of using AI in teaching medical students.

Areas of potential use included as a source of literature and an extensive information pool for research. Some positive points given included standardization of evaluation, helpfulness in active learning, and scenario generation, training organization, and case simulation abilities. It can support continuous evaluations and, to a lesser extent, formative assessment. Respondents generally believed that AI applications would redefine every aspect of learning, make things easier, help standardize patient care, and allow students to perform clinical procedures in a safe environment. One student commented: “It can help us at the skill lab” and another mentioned: “It can help us prepare for clinical exams”.

When asked if they think AI will replace some specialties in healthcare shortly, most respondents (82.25%) disagreed. Respondents believed AI would aid specialties like patient triage, radiology, and pathology. The general agreement was that AI can help but it cannot replace a specialty, and the need for human supervision and clinical sense would remain. As one faculty member said, “Humans cannot be replaced by AI”.

Faculty and Students Raised Ethical Concerns About the Use of AI in Medical Education

Many respondents thought that AI could threaten academic integrity and that sources of information and accuracy were sometimes questionable. Another concern was plagiarism and not using own thoughts, which led to ownership issues. They believed that the use of AI lacks respect for intellectual property. One faculty member mentioned, “These thoughts are not genuine”, while a student said, “Questionable interpretation of information sometimes occurs with AI”.

Privacy and confidentiality issues might arise with the use of AI in healthcare. Another concern was that AI was not culture-sensitive and was mainly based on Western values and cultures. Another concern raised by a student was that “It is a tool that can be misused”.

Faculty and Students Have Different Perspectives on Resource Availability, Curriculum Adaptability, and the Need for Training to Incorporate AI in Medical Education at Jazan University 

Regarding resources available for AI use at the Faculty of Medicine, faculty members and students had conflicting views; most faculty members (n=7, 63.63%) thought that resources are adequate, while most students (n=29, 85.3%) thought not. However, faculty members also thought there was a need for human resource experts and software updates, and that the Faculty needed to invest more in AI technology.

Regarding the need for training on AI use, faculty and students (n=36, 80%) felt there was a need for training although most thought that faculty members need more training. As one faculty member described students: “They are already better than us in using this technology and it comes naturally to them”.

Also, both faculty and students (n=29, 64.44%) thought that the curriculum could adapt to AI integration into medical education at the Faculty. However, some faculty members believe that minor modifications in course descriptions might be needed, and there was also a need for regulation and adaptation. 

## Discussion

The qualitative study at the Faculty of Medicine, Jazan University, Saudi Arabia, offered valuable insights into the views of faculty staff and medical students' inclinations towards AI in medical education.

One of the main findings of the study lies in the clearly expressed positive opinion of AI as a valuable tool that holds great potential for renewing medical education. The more comprehensive discussion (91.11%) was that AI systems would play a positive role in medical education such as knowledge acquisition, teaching materials, diagnostic tests, and simulations/cases resembling an actual patient. In respondents' view, AI would have a three-fold opportunity: (i) to be regarded as a fountainhead of literary artifacts and a treasure trove of the information universe and to make assessments the same everywhere, (ii) creative teaching and the generation of scenarios, (iii) the organization of training and the simulation of cases. Hence, this concept agrees with the evidence of AI's contribution to improving medical education, as pointed out in other studies [4,6].

On the negative side, interviewees indicated an academic integrity threat, access to false information and information that is not accurate, plagiarism, and infringement of intellectual property rights. Furthermore, privacy and confidentiality issues were highlighted. These issues are a genuine concern to faculty members, especially when trying to maintain academic integrity while performing fair and valid student assessments. 

In addition, the issue of AI systems' lack of sensitivity to cultures was raised. AI systems are developed in a Western context, which might pose problems for contexts with different cultures. Although the literature is scarce in the area of cultural adaptability of AI systems, Samuel et al. [14] have suggested a framework that helps train learners from different cultural backgrounds on AI concepts. However, the opposite is also needed: AI systems that are relevant and sensitive to the cultural norms where they are used. Parag et al. argue that AI systems need to adapt to cultural diversity for better patient care, which is the ultimate goal of medical education [15]. 

Weidener and Fischer concluded in their article that ethics, privacy, and regulations need to be a part of AI use in medical education [16]. Chan et al.'s study also revealed the ethical challenges of implementing AI in medical education [6]. They focused on ethical judgment and the inability of AI to teach empathy. These worries are undeniable, and measures should be taken to ensure that ethical and responsible standards won't be compromised despite the need for AI in medicine [6,8].

The study also investigated whether resources are available, whether the curriculum can be tailored, and what training is required for Jazan University's AI implementation in medical education. Although there was a disagreement on the availability of human capital, staff and students acknowledged the power of hiring human resource experts, software with updates, and utilization of AI technology. Investment in this human and technical capital is needed. Besides, the faculty and students had the same opinion (80%) regarding the necessity of AI training. Moreover, a maximum of respondents (64.44%) felt the curriculum could be adopted quickly with some minor modifications for AI use across medical education. Still, some professors suggested that modifications, regulations, and adaptations are necessary.

The results of our study comply with the earlier studies that have proved that AI will bring positive feelings among the students of medicine and healthcare professionals, and putting together an AI training syllabus in medical education will be a mandatory task [8-10]. 

Limitations

The study identified significant factors, but it is also necessary to pinpoint the study's limitations. One limitation is that the study was conducted in one medical faculty in Saudi Arabia, which might limit the generalizability of the findings from pure instances of different societal, economic, and educational contexts. Another limitation of this work is that qualitative studies can give subjective results and interpretations. The latter can deliver qualitative data of rich flavor and great depth, which is not available in quantitative research, while the former is more objective and provides more generalizable results.

## Conclusions

The study conducted at the Faculty of Medicine, Jazan University, Saudi Arabia, gives critical information concerning academic members' and medical students' opinions and thoughts about adopting AI in medical education. The study's results unveiled a generally positive feeling about integrating AI into medical learning that can be used for research, knowledge acquisition, assessment, and simulation and that this integration will improve both the quality and efficiency of the learning experience. However, the study also highlighted several ethical concerns raised by faculty members and students, which include violation of academic integrity, wrong information accuracy, plagiarism, intellectual property issues, information privacy and confidentiality concerns, and insensitivity to the social culture of AI systems. These concerns are genuine and should be regulated before AI is incorporated into medical education to preserve ethics and responsibility.

Not only that, but the study highlights important perspectives of faculty and students related to investing in applying AI. Resources like hiring expert human resources in AI, software, and updates were thought needed to be adopted by the Faculty of Medicine. Further research on the best ways to utilize AI, as well as joint effort and planning, is mandatory to ensure that AI is used responsibly, to be a positive factor in education, and, ultimately, to improve the quality of patient management.
